# Expression and copper binding characteristics of *Plasmodium falciparum* cytochrome *c* oxidase assembly factor 11, Cox11

**DOI:** 10.1186/s12936-022-04188-5

**Published:** 2022-06-07

**Authors:** Abdulmalik Abdullahi Salman, J. P. Dean Goldring

**Affiliations:** Biochemistry, University of KwaZulu-Natal, Pietermaritzburg, 3201 South Africa

**Keywords:** Plasmodium, Malaria, Cox11, Cytochrome *c* oxidase, Copper

## Abstract

**Background:**

Copper is an essential metal for living organisms as a catalytic co-factor for important enzymes, like cytochrome *c* oxidase the final enzyme in the electron transport chain. *Plasmodium falciparum* parasites in infected red blood cells are killed by excess copper and development in erythrocytes is inhibited by copper chelators. Cytochrome *c* oxidase in yeast obtains copper for the Cu_B_ site in the Cox1 subunit from Cox11.

**Methods:**

A 162 amino acid carboxy-terminal domain of the *P. falciparum* Cox11 ortholog (*Pf*Cox11Ct) was recombinantly expressed and the rMBP*Pf*Cox11Ct affinity purified. Copper binding was measured in vitro and in *Escherichia coli* host cells. Site directed mutagenesis was used to identify key copper binding cysteines. Antibodies confirmed the expression of the native protein.

**Results:**

rMBP*Pf*Cox11Ct was expressed as a 62 kDa protein fused with the maltose binding protein and affinity purified. rMBP*Pf*Cox11Ct bound copper measured by: a bicinchoninic acid release assay; atomic absorption spectroscopy; a bacterial host growth inhibition assay; ascorbate oxidation inhibition and in a thermal shift assay. The cysteine 157 amino acid was shown to be important for in vitro copper binding by *Pf*Cox11whilst Cys 60 was not. The native protein was detected by antibodies against rMBP*Pf*Cox11Ct.

**Conclusions:**

*Plasmodium *spp. express the *Pf*Cox11 protein which shares structural features and copper binding motifs with Cox11 from other species. *Pf*Cox11 binds copper and is, therefore, predicted to transfer copper to the Cu_B_ site of *Plasmodium* cytochrome *c* oxidase. Characterization of *Plasmodium* spp. proteins involved in copper metabolism will help sceintists understand the role of cytochrome *c* oxidase and this essential metal in *Plasmodium* homeostasis.

## Background

Mitochondria with the associated electron transport chain are present in most apicomplexans, have small mitochondrial genomes and appear to be present throughout all the stages of their life cycle [1]. Their electron transport chains have sufficient differences to the electron transport chains of their mammalian hosts to make them promising drug targets [1, 2]. Human malaria caused by *Plasmodium* spp. parasites is the most prevalent apicomplexan infection. The parasite is passed by the bite of an infected mosquito in human infections to the blood of the human host, infects the liver and then red blood cells, which coincides with clinical symptoms. *Plasmodium* parasites in red blood cells depend predominantly on glycolysis for the generation of ATP and electron micrographs show the presence of a single mitochondrion [3, 4]. Despite a reduced dependence on mitochondria, the parasites are sensitive to drugs targeting the electron transport chain during this stage of development indicating the importance of the pathway for the parasite [1].

Proteins participating in the electron transport chain contain iron and copper to transport electrons leading to the subsequent generation of ATP. Copper is essential for most living organisms as the metal can cycle between the reduced (Cu^+^) and more stable oxidized (Cu^2+^) form under physiological conditions and so participate in redox reactions and electron transfer. Copper can be toxic due to its ability to undergo Fenton reactions leading to the generation of toxic reactive oxygen species [5]. Copper can be toxic, can displace metals in proteins, is rarely free in cells and is, therefore, tightly regulated and bound to proteins, like copper chaperones.

During the erythrocytic stage *Plasmodium* spp. can, like yeast and mammalian cells, import copper via the copper transport protein (Ctr1) expressed initially on the red cell membrane and then the parasitophorous vacuolar membrane [6]. The parasite also obtains copper from the digestion of ingested host erythrocyte Cu/Zn superoxide dismutase [7, 8]. *Plasmodium* spp. do not express an ATOX-1 (Antioxidant Protein 1) or CCS (Copper Chaperone for Superoxide dismutase) chaperone homologue that shepherd copper to ATPases in the Golgi apparatus or to Cu/Zn superoxide dismutase respectively in mammalian cells [9]. ATPases are involved in the export of copper from cells and *Plasmodium* express a copper P-ATPase [8] that is important for parasite fertility [10]. Excess copper is toxic for *Plasmodium* spp. [11] and chelating copper in in vitro growth media with neocuproine or tetrathiomolybdate inhibit the growth of intraerythrocytic parasites [12].

*Plasmodium* spp. mitochondria have the four complexes of the electron transport chain which includes the copper containing cytochrome *c* oxidase of complex IV [13]. Cytochrome *c* oxidase is assembled in many steps, requiring over 30 accessory proteins [14]. The Cox17 protein is a key participant in mitochondrial copper transport to proteins for insertion into cytochrome *c* oxidase [15]. *Plasmodium* spp. express Cox17 [16]. Cox17 was first observed in the cytoplasm and mitochondria of yeast which suggested a role in copper transport to mitochondria [17], but subsequent data using Cox17 tethered to the mitochondrial matrix in yeast does not support this suggestion [18]. Cox17 transfers copper to Sco1 and Cox11 which in turn insert copper into the Cu_A_ and Cu_B_ sites of Cox2 and Cox1 of cytochrome *c* oxidase [18, 19]. Copper appears to be transported to mitochondria by the phosphate carrier Pic2 in yeast [20] and SLC25A3 in mammalian cells [21].

Cox11 in eukaryotes is attached to the inner mitochondrial membrane via a single transmembrane domain with the N-terminus in the mitochondrial matrix and the C-terminus in the intermembrane space [22–24]. The protein metalates the Cu_B_ site of Cox1 with copper in cytochrome *c* oxidase [25]. Cox11 copper is coordinated by two cysteines in a CFCF motif found in the C-terminal domain [23]. Cox11 is essential in yeast for the assembly of cytochrome *c* oxidase and the activity of cytochrome *c* oxidase in *Arabidopsis* [23, 26]. The present study extended the understanding of *Plasmodium* spp. copper metabolism and the synthesis of cytochrome *c* oxidase by studying recombinant *P. falciparum* Cox11 and showing that the recombinant protein binds copper in vitro and in vivo when expressed in *Escherichia coli* host cells.

## Methods

### Ethical clearance

All procedures involving animals were approved by the University of KwaZulu-Natal, Animal Research Ethics Committee (approval number: 0045/15/Animal).

### Identification of the gene and analysis of *Plasmodium* spp. Cox11 protein sequences

*Plasmodium falciparum* cytochrome *c* oxidase assembly protein *Pf*Cox11 (PF3D7_1475300) was identified [6] using a BLASTp search of the Plasmodb.org database with the Human Cox11 protein amino acid sequence (UniProtKB-Q9Y6N1 COX11_HUMAN).

**Fig. 1:**
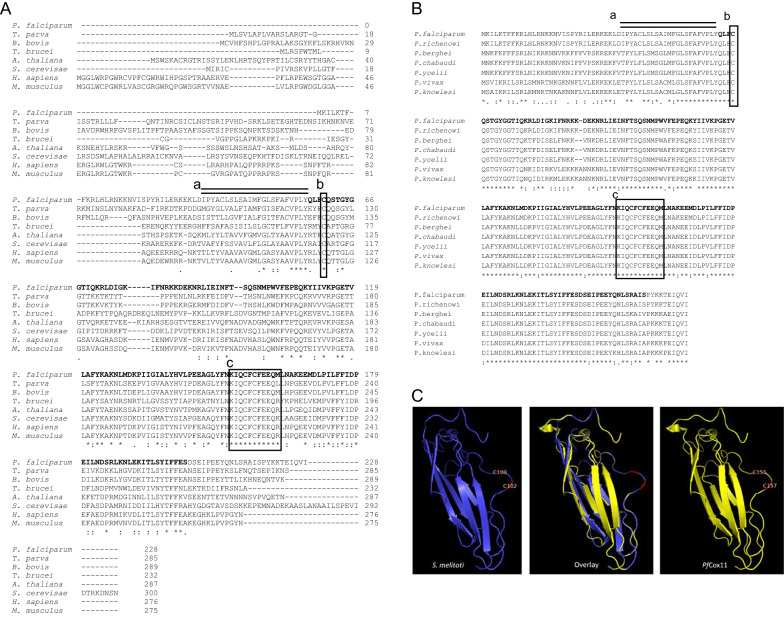
Analysis of *Plasmodium* spp. Cox11 amino acid sequences. The *P. falciparum* Cox11 amino acid sequence was aligned with Cox11 from **A** *Theileria, Babesia, Trypanosoma, Arabidopsis, Saccharomyces, Homo* and *Mus* sequences and **B** *Plasmodium reichenowi, Plasmodium berghei, Plasmodium chabaudi, Plasmodium yoelii, Plasmodium vivax,* and *Plasmodium knowlesi.* Key: a: *P. falciparum* Cox11 transmembrane domain, b: conserved cysteine (*P. falciparum* C60) and c: conserved CFCF motif (*P. falciparum* C155 and C157). The cloned *P. falciparum* sequence is in bold script. **C** for comparative purposes the protein model for *Sinorhizobium meliloti* Cox11 (PDB:ISO9) amino acid sequence served as a template (left panel) for homology modelling of the *P. falciparum* Cox11 amino acid sequence (right panel) and the two models superimposed (middle panel). The *S. meliloti* and *P. falciparum* Cox11 Cys-100/102 and 155/157 positions in the conserved CFCF sequence are illustrated

Transmembrane domains in the *Pf*Cox11 amino acid sequence (PF3D7_1475300) were identified using the TMHMM 2.0 programme [27]. Multiple sequence alignment was generated with Clustal Omega (http://ebi.ac.uk/Tools/msa/clustalo) [28]. Theoretical molecular weight and isoelectric point were determined using PROTPARAM (http://web.expasy.org/protparam). The *P. falciparum* Cox11 structure (Fig. 1) was modelled on the *Sinorhizobium meliloti Sm*Cox11 homologue NMR structure template (PDB: 1so9) template [24] using the Swiss-Pdb DeepView program [29].

### PCR amplification, mutagenesis and cloning of the C-terminal domain of *Plasmodium falciparum* PfCox11 and mutants

The *PfCox11* gene portion coding for the predicted C-terminal domain and equivalent to that found in the mitochondrial intermembrane space for the yeast protein [22] was amplified using the following primers: r*Pf*COX11Ct-fwd cc**GTCGAC**CAATTATTTTGTCAATCCACAGG and r*Pf*COX11Ct-rev 5' tt**CGTCAG**TCAGGAAATAVGCCTTGAGG with added Sal1 and Pst1 restriction endonuclease sites shown in bold. Primers for site-directed mutagenesis [30] for the C60A mutation were r*Pf*COX11Ct_C60A_-F1 GAAAGACGCGCAGACTAATTCGAGCTC, r*Pf*COX11Ct_C60A_-R1 GGCCAGTGCCAAGCTTGCCTGCAG, and r*Pf*COX11Ct_C60A_-F2 GTCG-ACCAATTATTTGCGCAATCCACAGG, r*Pf*COX11Ct_C60A_-R2 CTGTGGATTGCGCAAATAAT-TGGTCGAC. The C157A mutant was amplified using the following primers: r*Pf*COX11Ct_C157A_-F1 GAATTCGGATCCTCTAGAGTC r*Pf*COX11Ct_C157A_-R1 TGCCAAGCTTGCCTGCAG and r*Pf*COX11Ct_C157A_-F2 CAATGTTTTGCCTTTGAAGAAC, r*Pf*COX11Ct_C157A_-R2 GTTCTTCA-AAGGCAAAACATTG. The polymerase chain reaction was conducted with a reaction mix (20 µl) containing 1–100 ng template DNA, 0.5 µM of each primer, 2.5 mM MgCl_2,_ 0.2 mM dNTPs, 1 U of Phusion DNA polymerase (New England Biolabs) for PCR and site directed mutagenesis, and 1× Taq buffer in a T100^™^ thermocycler (Bio-Rad, USA). An initial denaturation step (94 °C; 3 min), followed by 35 cycles of denaturation (94 °C; 30 s), annealing (temperature determined by the Tm of the primers, 30 s), elongation (72 °C; 60 s) and final elongation (72 °C; 7 min) steps. Amplicons of the *Pf*Cox11-Ct were cloned into the pGEMTeasy vector and the plasmid propagated in JM109 *E. coli* host cells. The plasmid with insert was isolated and digested with Sal1 and Pst1 restriction endonucleases. The insert was gel purified and ligated in frame with an N-terminal maltose binding protein (rMPB) in the pMal-c2x expression vector digested with the same two restriction endonucleases (New England Biolabs, USA). The sequence of the cloned gene was confirmed by DNA sequencing (Central Analytical Facility, Stellenbosch University, South Africa). The mutated gene fragments were PCR amplified, digested with Sal1and Pst1 restriction endonuclease and ligated into the pMal-c2x vector as described above.

### Expression and purification of recombinant MBPPfCox11Ct and mutants

Recombinant pMal-c2c vectors containing the *PfCox11Ct* insert were transformed into competent *E. coli* BL21 host cells. A single *E. coli* transformant was picked from an agar plate and grown overnight (16 h) in 10 ml 2 × YT medium (30 °C, 200 rpm) containing 100 µg/ml ampicillin. A 1:100 dilution of the culture was added to 400 ml 2 × YT medium with 100 µg/ml ampicillin and grown (30 °C, 200 rpm) to an OD_600_ between 0.5 and 0.6. Recombinant protein expression was induced with 0.5 mM IPTG with additional ampicillin. The bacterial cells were pelleted by centrifugation (4000×*g*, 4 °C, 10 min) and the supernatant discarded. The pellet was resuspended in 10% of the original culture volume of buffer (20 mM Tris–HCl, 200 mM NaCl, 1 mM EDTA, pH 7.4) and lysed with 9 freeze–thaw cycles (−196 °C and 37 °C) followed by sonication on ice (3 cycles, 30 s/burst, 30 s on ice between sonication). The lysates were centrifuged (12,000×*g*, 4 °C, 25 min). The supernatant was added to a 1 ml amylose resin (New England Biolabs, USA) equilibrated with Tris–HCl buffer (20 mM Tris, 200 mM NaCl, pH 7.4) and was washed with wash buffer (100 mM Tris, 200 mM NaCl, pH 7.4). Bound protein was eluted with the wash buffer containing 0.3 mM maltose and eluted fractions were collected and concentrated with an Amicon Ultra-15 Centrifugation Filter Unit (10 kDa MWCO) by centrifugation (5000×*g*, 4 °C) [31, 32]. Samples were dialysed at 4 °C against 3 changes of 100 mM Tris–HCl buffer pH 7.4. The Bradford dye binding assay [33] was used to estimate the protein concentration of samples as adapted [34]. Proteins were evaluated on a 12.5% reducing SDS-PAGE gel stained with Coomassie Blue [35].

### BCA copper release assay

Purified recombinant rMPB-*Pf*Cox11Ct and mutants were tested for their ability to bind copper after purification (in vitro) and during expression (in vivo). *Escherichia coli* cells expressing the protein intended for in vitro or in vivo copper binding studies were grown in a medium containing 0 or 0.5 mM CuCl_2_ respectively, added during induction of protein expression [16, 36]. The protein was affinity purified as described above. In the in vitro studies*,* 10 µM purified rMBP*Pf*Cox11Ct was incubated with 20-fold molar excess of CuCl_2_ (15 min; 23 °C). Ascorbate (0 or 10 mM) was included in the reaction mix to reduce free copper. Excess unbound copper was removed by dialysis (2 × 2 h; 23 °C followed by 16 h; 4 °C). The dialysed sample was mixed with 30% (w/v) trichloroacetic acid (3:1 v/v) and centrifuged (12,000×*g*; 2 min; 23 °C). Released copper was detected by mixing with bicinchoninic acid (BCA) solution (0.15 mM BCA, 0.9 M NaOH, 0.2 M HEPES) with or without 2 mM ascorbate and the absorbance read at 354 nm [37]. To chelate copper, and thus render it unavailable for binding to a copper binding protein, EDTA was added to the BCA release assay prior to the addition of copper. In the in vivo assay, 10 µM purified rMBP*Pf*Cox11Ct grown in the presence of copper was affinity purified and added to the BCA copper release assay.

### Atomic absorption spectroscopy

The amount of copper bound to the recombinant proteins was quantified by atomic absorption spectroscopy (AAS) using an Agilent Varian AA280FS atomic absorption spectrophotometer. The reference solution was a certified atomic absorption standard (Fischer Scientific Co.) prepared at 1.0 ± 0.01 mg/ml in dilute nitric acid. Samples were prepared as described for the in vitro copper binding assessment using the BCA release assay.

### Growth of *E. coli* bacteria expressing recombinant proteins in the presence of toxic concentrations of copper

The influence of rMBP*Pf*Cox11Ct on the growth of *E. coli* host cells in the presence of toxic concentrations of copper was determined. Overnight cultures of *E. coli* cells alone or expressing the recombinant MBP or rMBP*Pf*Cox11Ct proteins were grown in 2 × YT medium (30 °C, 200 rpm) containing ampicillin (100 μg/ml). At an OD_600_ between 0.5 and 0.6, recombinant expression was induced with 0.5 mM IPTG and additional ampicillin (100 μg/ml) and 0 or 8 mM copper added and bacterial growth (30 °C, 200 rpm) monitored at OD_600_ for 6 h.

### Inhibition of copper-catalysed ascorbate oxidation assay

Copper catalyses the oxidation of ascorbate in vitro, and the ability of rMBP*Pf*Cox11Ct and mutants to bind copper and inhibit the ascorbate oxidation reaction was assessed. The pH of a 2 mM ascorbate stock solution was adjusted to pH 4.5 with dilute NaOH. The reaction mixture contained 5 µM protein, 120 µM ascorbic acid and 8 µM CuCl_2_ (1 ml total volume). Recombinant proteins were added to the reaction mixture and the absorbance of the solution at 255 nm was followed for 300 s at 25 °C [6, 38].

### Differential scanning fluorescence to determine rMBPPfCox11Ct thermal melt temperature

The assay described by Niesen et al. [39] with minor modifications was followed. Protein (0.5 μg) in phosphate buffer pH 7.4 and 10 × SYPRO Orange in 25 μl. The assay was run in Xtra-clear qPCR tubes (Star labs) in a Rotor-Gene 6000 RT-PCR machine (Corbett). The condition parameters set were high resolution melt (HRM) which has an λ excitation at 470 nm and an λ emission at 570 nm, which was used to measure the SYPRO Orange fluoresce probe. A temperature range of 25–90 °C was used with a ramp temperature of 0.3 °C/s. The change in fluorescence/change in temperature (dF/dT) was calculated and the dF/dT data was plotted against temperature and the highest dF/dT point on each peak was referred to as the T_m_.

### Antibody production

The amino acid sequence of rMBP*Pf*Cox11Ct was used to identify immunogenic epitopes with Predict7 software [40]. The peptide sequence KIQF(Abu)F(Abu)EEQMLNAKEEM where internal cysteines were replaced with alpha aminobutyric acid (Abu), was synthesized by GeneScript, USA. The C-terminal cysteine residue was added for *m*-maleimidobenzoyl-N-hydroxysuccinimide ester (MBS) coupling to rabbit serum albumin [41]. Two Hy-line brown laying hens were immunized with the peptide-carrier conjugate equivalent to 200 µg peptide or 50 µg affinity purified rMBP*Pf*Cox11Ct per immunization, emulsified with Freund’s complete adjuvant for the first immunization and Freund’s incomplete adjuvant for three subsequent immunizations at 2-week intervals. Chicken IgY from the yolks of eggs collected 4–16 weeks after the first immunization, was isolated using the PEG6000 precipitation method [42]. The anti-peptide IgY and anti-rMBP*Pf*Cox11Ct IgY was affinity purified using a peptide or rMBP*Pf*Cox11Ct affinity column prepared by coupling the respective molecules to a Sulfolink^™^ or Aminolink^™^ resin according to the manufacturer’s instructions (Thermo Fisher Scientific).

### *Plasmodium berghei* parasites

*Plasmodium berghei* parasites (donated by P. Smith University of Cape Town, originally from A.P. Waters Glasgow University) were propagated in male BALB/c mice by intraperitoneal injection of the parasite stabilate (1 × 10^7^ parasitized mouse red blood cells) [43]. Parasitaemia was monitored daily on a Giemsa-stained thin blood smear of tail blood [44]. Once parasitaemia reached a suitable level, mice were bled, and the blood collected in a heparinized vacuum test tube.

### SDS-PAGE and western blotting

Native and recombinant proteins were detected with affinity purified anti-peptide antibodies in a western blot [45]. Blood from infected mice was washed twice in PBS and resuspended in lysis buffer (20 mM Tris–HCl, 10 mM Na_2_EDTA, 1% (v/v) Triton X-100, pH 7.4). The lysates (approximately 10^5^ parasites) and recombinant proteins were separated on a 12.5% reducing SDS-PAGE gel, transferred to a nitrocellulose membrane and blocked by incubation in 0.5% (w/v) non-fat milk (Amresco, USA) in Tris-buffered saline (TBS, 20 mM Tris–HCl, 200 mM NaCl, pH 7.4). The membrane was incubated in an appropriate dilution of primary antibody followed by the secondary antibody diluted in 0.5% (w/v) BSA in TBS. The blots were developed by incubation in either 0.06% (w/v) 4-chloro-1-naphthol/0.0015% (v/v) H_2_O_2_ or Pierce^™^ enhanced chemiluminescence (ECL) western blotting substrate. Primary antibodies were affinity purified chicken anti- KIQF(Abu)F(Abu)EEQMLNAKEEM peptide IgY (2.5 µg/ml), chicken anti-r*Pf*Cox11Ct protein IgY (1 µg/ml) or mouse monoclonal anti-MBP IgG (1:12 000) (New England Biolabs, USA). Detection antibodies were rabbit anti-chicken IgY-horse radish peroxidase (HRPO) conjugate (1:5000) (Jackson Immuno-Research Laboratories, USA) or goat anti-mouse IgG-HPRO conjugate (1:5000) (Roche, Germany).

## Results

### Bioinformatics

Choveaux et al. [6] identified a putative Cox11 using the human Cox11 amino acid sequence as the input sequence, alongside 13 copper-dependent protein orthologues in the *P. falciparum* genome from a BLASTp search of the PlasmoDB genome database [46]. The *Plasmodium* Cox11 sequences lack the 51–61 amino acid N-terminal extension found in the *Theileria, Babesia, Arabidopsis, Homo* and *Mus* sequences and as a result have 4, instead of the 5 or 6 cysteines seen in the plant, yeast or mammalian Cox11 sequences (Fig. 1a). The *P. falciparum Cox11* gene is found on chromosome 14, and codes for a 228 amino acid protein sequence [47]. The *Plasmodium* spp. sequences, like all other characterized Cox11 sequences, have a 19 amino acid transmembrane domain (Fig. 1a, b) followed by a conserved cysteine (Cys-60) and 94 amino acids towards the C-terminus a conserved CFCF motif (Cys-155 and Cys-157, Fig. 1a, b). There is an 11 amino acid region around the CFCF motif that includes Lys-152 that is 100% conserved in multiple species (Fig. 1a). These three cysteines and the lysine are essential for yeast Cox1 activity [48]. *Plasmodium falciparum* also contains a conserved tyrosine (*Pf* Tyr-139) while the hydrophobic valine found in the *Saccharomyces cerevisiae* (Val-226) homologue [48] is replaced with an isoleucine (*Pf* Ile*-*173). The *P. falciparum* sequence shares 44% identity with the human sequence and 71% identity with other *Plasmodium* spp. Cox11 sequences. The protein has a predicted molecular mass of 26.807 kDa, a pI of 8.71 [47] and the amino acid sequence is predicted to target the protein to the mitochondrial inner membrane [47] (http://busca.biocomp.Unibo.it/deepmito).

Expression profiling during the different stages of the *P. falciparum* life cycle showed that *Pf*Cox11 and *Pf*Cox1 are expressed during the sporozoite, trophozoite and gametocyte stages of parasite development [49] and interestingly levels of Cox11 peak 30 h and Cox1 40 h post-erythrocytic invasion [50]. Plasmodium spp. Cox11 proteins share the classical structural domains and functional motifs found in well-characterized Cox11 proteins from other species.

### Homology model

The NMR-solved structure of *S. meliloti* Cox11 (PDB:ISO9) [24] served as the template to generate a *P. falciparum* Cox11 (*Pf*Cox11) homology model (Fig. 1c). Banci et al. [24] describe the structure as having an immunoglobulin-like fold consisting of 10 β strands organized into a β-barrel. The two *P. falciparum* cysteines (*Pf* Cys-155 and Cys-157) in the CFCF sequence within the highly conserved 11 amino acid motif are on a surface loop on the structure shown in the diagram (Fig. 1c). The two cysteines are also located on a surface loop in the Alphafold predicted *P. falciparum* Cox11 protein model (https://www.uniprot.org/uniprot/Q8IK85). The Cys-60 amino acid residue is in the N-terminus of the protein and was not included in the *S. meliloti* model (PDB:ISO9) [24].

### Recombinant expression of rMBPPfCox11Ct and detection with anti-MBP antibodies

**Fig. 2:**
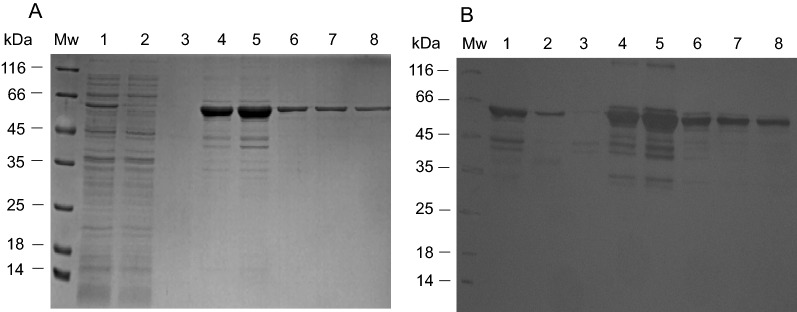
Evaluation of rMBP*Pf*Cox11Ct on SDS-PAGE and detection with anti-MBP antibodies on a western blot. Proteins were separated on a 12.5% reducing SDS-PAGE gel and stained with Coomassie blue (**A**) and (**B**) the same proteins on an identical gel electrophoretically transferred to nitrocellulose and probed with mouse anti-MBP monoclonal antibody and detected with goat anti-mouse IgG-horse radish peroxidase (HRPO) conjugate and enhanced chemiluminescence (ECL). Mw: molecular weight marker; lane 1, soluble *E. coli* lysate, and fractions from an amylose affinity matrix: unbound (lane 2); wash (Lane 3) and eluted in the presence of maltose (lanes 4–8)

The sequence coding for 162 amino acids of the carboxy-terminal domain of the *PfCox11* gene was cloned and expressed as a recombinant maltose binding fusion protein and called “rMBP*Pf*Cox11Ct”. A His tag fusion protein was insoluble and was not used. The identity of the cloned sequence was confirmed by DNA sequencing. The recombinant MBP fusion protein was affinity purified from *E. coli* host cell lysates using an amylose affinity matrix (Fig. 2a). A 62 kDa protein that corresponds to the 62.745 kDa, predicted from the cloned gene sequence, and consists of 19 kDa of *Pf*Cox11 and 43 kDa of the maltose binding protein fusion partner, was expressed and detected in a western blot by anti-MBP antibodies (Fig. 2b). Truncated versions of the recombinant protein, including a prominent 45 kDa protein, corresponding to the size of the rMPB fusion partner, were also detected by the anti-MBP antibodies. About half a milligram of protein (0.49 mg) was obtained from each gram of wet bacterial pellet (Table 1).

**Table 1:** Purification table for rMBP*Pf*Cox11Ct

Sample	Total vol (ml)	Total protein^a^ (mg)	Yield (%)	Yield (mg/g pellet)
Soluble *E. coli* lysate^b^	30.15 ± 1.91^c^	122.41 ± 2.20	100	42.07 ± 0.76
Unbound affinity column fraction	27.46 ± 2.06	103.33 ± 0.70	84.41 ± 2.09	35.51 ± 0.24
Washes	58.50 ± 7.78	11.08 ± 1.10	9.04 ± 0.74	3.81 ± 0.38
Affinity purified rMBP*Pf*Cox11Ct	23.59 ± 1.47	1.43 ± 0.19	1.17 ± 0.18	0.49 ± 0.06

^a^Protein determined by the Bradford protein assay (see Expression and purification of recombinant MBPPfCox11Ct and mutants)

^b^Original cell pellet = 2.91 ± 0.22 g

^c^Data presented are Mean ± SD values from duplicate purifications

### rMBPPfCox11Ct and mutants bind Cu^+^ in vitro

**Fig. 3:**
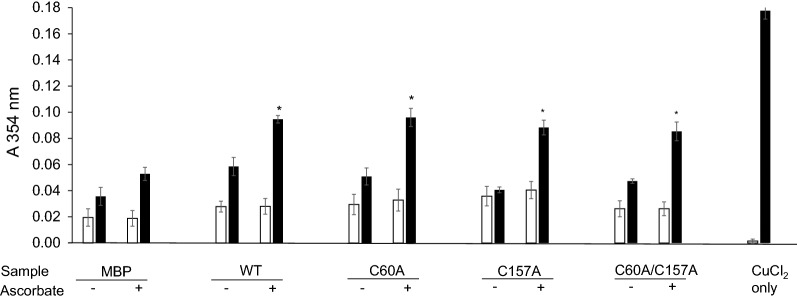
Copper binding to rMBP*Pf*Cox11Ct and mutants in vitro measured by the BCA release assay. Affinity purified rMBP*Pf*Cox11Ct or mutants C60A, C157A, C60A–C157A or rMPB were incubated with CuCl_2_ in the absence (−) or presence ( +) of ascorbic acid in vitro*.* Copper was detected with the BCA release assay without (open bars) or with (closed bars) the addition of ascorbic acid. Copper chloride was equimolar to protein. Results are the means ± SE of triplicate measurements from duplicate samples. A two-way ANOVA with Bonferroni multiple comparison tests were conducted on the results of samples incubated with CuCl_2_ in the presence of ascorbate. Comparisons are between rMBP*Pf*Cox11Ct and mutants and rMPB where *P < 0.001

Recombinant copper chaperones, *Pf*Cox17 and the C-terminus of recombinant *Pf*Ctr-1, along with copper chaperones from different sources have been shown to bind copper in vitro*.* Purified rMBP*Pf*Cox11Ct was exposed to both Cu^+^ and Cu^2+^ in vitro before the protein was denatured and the oxidative state of the released Cu determined by the BCA assay in the presence or absence of the reducing agent, ascorbate. The rMBP*Pf*Cox11Ct bound cuprous ions (Fig. 3). To identify which particular cysteine residues are involved in copper binding, the *Pf*Cox11 Cys-60 and Cys-157 residues were substituted with alanine by site-directed mutagenesis [51]. Single C60A, and C157A mutants and a double C60A/C157A mutant were generated. The amino-acid sequences of the mutants were confirmed by sequencing the respective DNA sequences in the host plasmid. The remaining cysteine (Cys-155) was not mutated as the DNA sequence around the cysteine is AT rich and primer sequences would lack specificity for the targeted site in the experimental approach used. There was no significant difference in the binding of copper in vitro by the rMBP*Pf*Cox11Ct protein or its derivatives containing the three different mutations in the BCA release assay (Fig. 3). The proteins containing the C157A and the C60A/C157A double mutations, like the wild type protein, bound copper in the assay, but appeared to bind marginally less copper than the wild type protein (Fig. 3). The rMPB fusion partner did bind some copper, as has been described before, and was included as a control in all experiments [6].

### EDTA inhibition of copper binding and detection of copper with atomic absorption spectroscopy

**Fig. 4:**
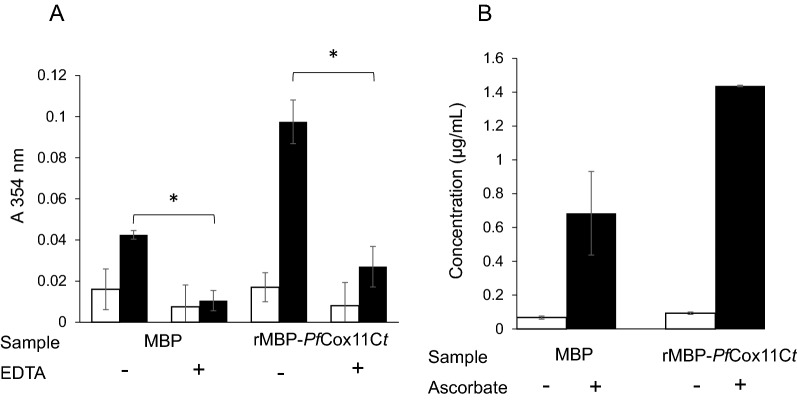
Copper binding to rMBP*Pf*Cox11Ct is inhibited by EDTA and measured by atomic absorption spectroscopy. Affinity purified rMBP*Pf*Cox11Ct or rMPB were incubated with and without EDTA and with CuCl_2_ in vitro*.* Copper was detected with the BCA release assay without (open bars) or with (closed bars) the addition of ascorbic acid. Results are the means ± SE of triplicate measurements from duplicate samples. A two-way ANOVA with Bonferroni multiple comparison tests were conducted on results of samples incubated with CuCl_2_ in the presence of ascorbate. Comparisons are between rMBP*Pf*Cox11Ct or rMPB without and with EDTA, *P < 0.001. **A**, **B** Affinity purified rMBP*Pf*Cox11Ct or rMPB was incubated with CuCl_2_ in the absence (open bars) or presence (solid bars) of ascorbic acid and bound copper was quantified using atomic absorption spectroscopy at 324.5 nm. Data is the mean ± SD of duplicate samples.

EDTA has been reported to chelate copper and remove copper from the media and the chelated copper is unavailable to bind to molecules [52]. Therefore, it was expected that copper chelated to EDTA would no longer be available for binding to the rMBP*Pf*Cox11Ct. The binding of Cu^+^ to rMBP*Pf*Cox11Ct was inhibited by EDTA as measured by the BCA-release assay (Fig. 4a). The binding of copper measured by the BCA-release assay was confirmed (Fig. 4b) with atomic absorption spectroscopy [37]. The rMPB fusion partner bound small amounts of copper as seen in the assays above. All the above assays indicate both rMPB and to a greater extent rMBP*Pf*Cox11Ct bound Cu^+^.

### Binding of copper to rMBPPfCox11Ct and rMBPPfCox11Ct mutants in vivo in *E. coli* host cells

The ability of rMBP*Pf*Cox11Ct and the protein with mutated cysteine residues to bind Cu^+^ in an in vivo cellular environment was evaluated with two different approaches. Initially, *E. coli* host bacteria with plasmids containing the *PfCox11Ct* DNA sequence or the three mutated sequences were grown in the presence of CuCl_2_ at non-toxic concentrations of copper and recombinant fusion protein expression induced, followed by protein isolation. Copper binding to rMBP*Pf*Cox11Ct expressed in copper rich medium was determined with the BCA-release assay. In the second approach the plasmid containing host cells were grown in the presence of increasing levels of copper and host bacteria growth patterns monitored.

**Fig. 5:**
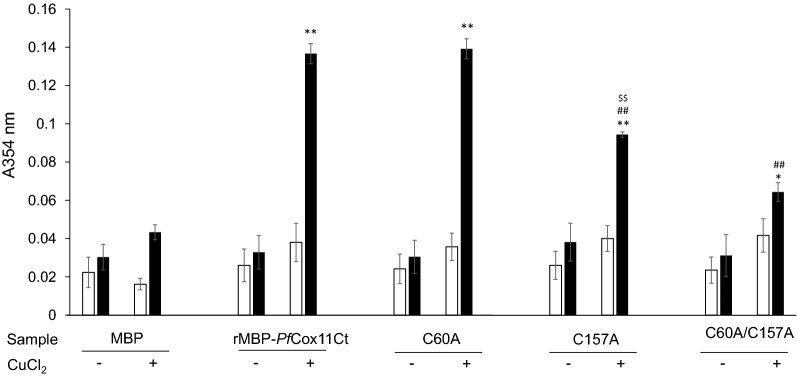
Copper binding to rMBP*Pf*Cox11Ct and mutants in vivo measured by the BCA release assay. *E. coli* host cells expressing rMBP*Pf*Cox11Ct or the mutants C60A, C157A and C60A/C157 or rMPB were grown in the presence of 0.5 mM CuCl_2_. The expressed protein was affinity purified and copper was detected with the BCA release assay without (open bars) or with (closed bars) the addition of ascorbic acid. Results are the means ± SE of triplicate measurements from duplicate samples. A two-way ANOVA with Bonferroni multiple comparison tests were conducted on the results comparing: rMBP*Pf*Cox11Ct and mutants wrt rMPB *P < 0.05 and **P < 0.001, rMBP*Pf*Cox11Ct wrt C157A and C60A/157A ^##^*P* < 0.001, C157A wrt C60A/C157A ^$$^*P* < 0.001

rMBP*Pf*Cox11Ct isolated from *E. coli* grown in the presence of CuCl_2_ bound copper in the in vivo copper enriched environment. The bound copper was in the Cu^+^ oxidation state, as inferred from the BCA release assay in the presence and absence of ascorbic acid (Fig. 5). Unlike in the in vitro BCA release assay (Fig. 3), there was more copper bound to the rMBP*Pf*Cox11Ct and there was a significant difference in copper binding between the rMBP*Pf*Cox11Ct native sequence and the proteins with mutated cysteines. The rMBP*Pf*Cox11Ct containing the C60A mutation, bound the same levels of copper as the protein without the mutation (Fig. 5). However, the rMBP*Pf*Cox11Ct protein with the C157A mutation bound significantly less copper and the protein with the double C60A/C157A mutant bound the least amount of copper (Fig. 5).

**Fig. 6:**
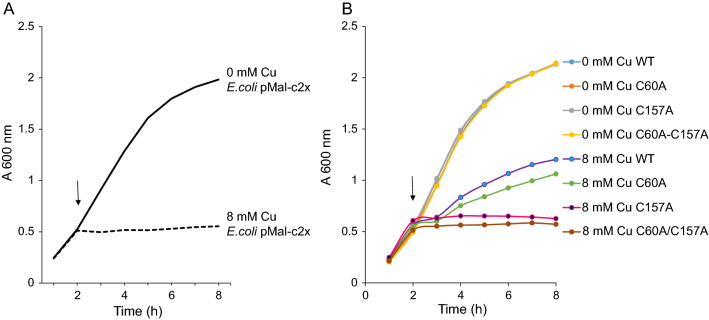
Growth of *E. coli* host cells expressing rMBP*Pf*Cox11Ct and mutants in the presence of copper. *E. coli* (BL21) growth was monitored at OD_600_ in the absence or presence of toxic concentrations of copper. **A** *E. coli* (BL21) expressing pMal-c2x. **B** *E. coli* (BL21) expressing rMBP*Pf*Cox11Ct, or the single C60A, C157A or double C60A/C157A mutant. Arrows indicate the addition of copper and IPTG to the growth media

In the second assay 8 mM copper chloride inhibited the growth of *E. coli* without a plasmid as seen by a plateau in the *E. coli* growth curve after copper addition (Fig. 6a). When expression of rMBP*Pf*Cox11Ct was induced it was proposed that the protein would bind copper and enable the host bacteria to continue to grow in the presence of otherwise toxic levels of CuCl_2_. The bacteria expressing rMBP*Pf*Cox11Ct continued to divide and grow, though at a lower rate compared to bacteria in the absence of CuCl_2_ (Fig. 6b). When bacteria expressing the rMBP*Pf*Cox11Ct protein with the C60A mutant were exposed to 8 mM CuCl_2_ like the non-mutant (wild type) sample, they grew but at a lower rate than the non-mutant. Bacteria expressing the rMBP*Pf*Cox11Ct protein with the single C157A or the double C60A/C157A mutation stopped dividing, indicating that the mutants did not bind copper and did not rescue the host cells in the presence of these toxic concentrations of copper (Fig. 6b).

The data from the two in vivo experiments suggests that Cys-60 appears not to be involved in the binding of copper, whilst Cys-157 appears to be involved in copper binding.

### Analysis of copper binding by rMBPPfCox11Ct using the rate of ascorbate oxidation

**Fig. 7:**
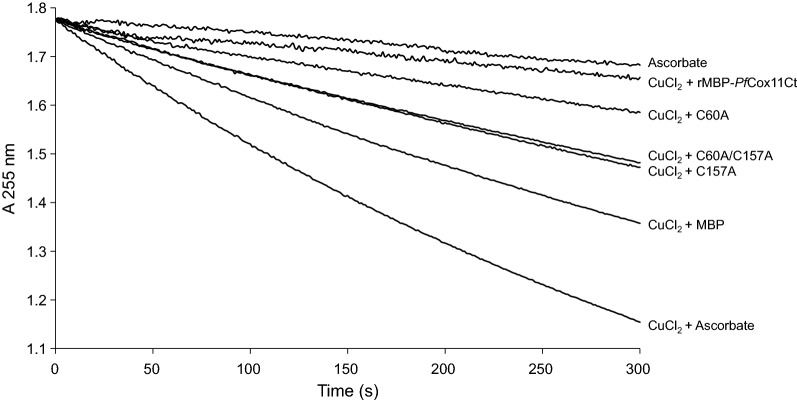
rMBP*Pf*Cox11Ct and mutants inhibit the copper-catalyzed oxidative degradation of ascorbate. Ascorbate alone and copper catalyzed ascorbate oxidation was followed by measuring absorbance at 255 nm. Inhibition of copper catalyzed ascorbate oxidation was determined by adding rMBP*Pf*Cox11Ct or the mutants C60A, C157A and C60A/C157A to the assay before addition of CuCl_2_

Proteins binding copper in vitro can influence the rate of copper-catalyzed oxidation of ascorbate and this has been used to determine copper binding by *Pf*Cox17, *Pf*Ctr-1 and other proteins [6, 16, 37]. The rate of copper-catalyzed ascorbate oxidation was monitored at 255 nm in the presence or absence of the native and mutated rMBP*Pf*Cox11Ct proteins. The addition of 8 mM CuCl_2_ caused a rapid oxidation of ascorbate indicated by a decrease in absorbance at 255 nm (Fig. 7). Oxidation of ascorbate was inhibited by rMBP*Pf*Cox11Ct and inhibited to a similar extent by the C60A mutant protein. Both the C157A and the C60A/C157A mutant proteins inhibited ascorbate oxidation to a lesser extent than the native rMBP*Pf*Cox11Ct protein. The inhibition of ascorbate oxidation is interpreted as the binding of copper by the proteins. These results follow the same pattern as the growth inhibition assay and support the importance of the Cys-157 for the binding of copper by *Pf*Cox11.

### Thermal shift analysis of rMBPPfCox11-Ct

**Fig. 8:**
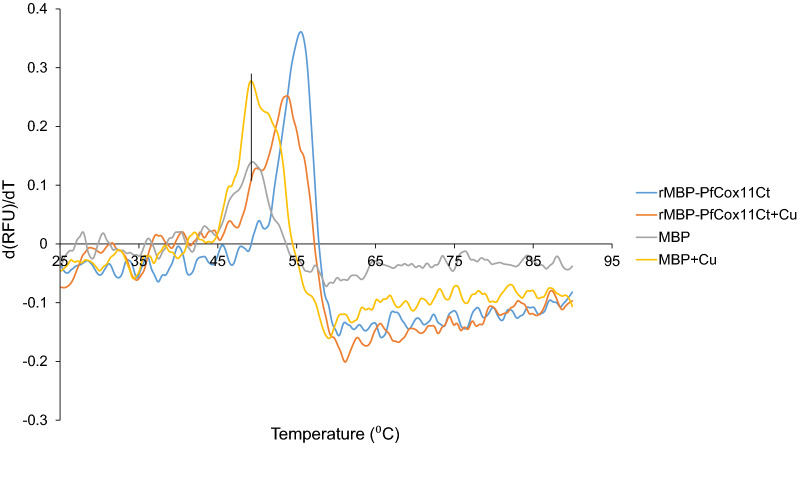
The effect of copper on the differential scanning fluorimetry first derivative of the fluorescence profile of rMBP*Pf*Cox11Ct. rMBP*Pf*Cox11Ct or rMPB with or without bound copper in phosphate buffer pH 7.4 was incubated with SYPRO® orange. The raw fluorescence data was measured from 25 to 90 °C and the first derivative calculated and presented. One of three different experiments with identical peaks is presented

Thermal shift analysis measures the thermal denaturation of a protein [53]. The denaturation profile of a protein can be influenced by the presence of metal ions, drug–protein or protein–protein interactions [39]. The differential scanning fluorescence (DSF) analysis presented in Fig. 8 showed a small decrease in the T_m_ of rMPB alone in the presence (49.12 ± 0.25 °C) or absence of copper (49.61 ± 0.35 °C). There was a more pronounced decrease in the T_m_ of rMBP*Pf*Cox11Ct in the presence of copper from 55.68 ± 0.12 °C to 52.42 ± 0.49 °C, a ΔT_m_ of −2.26 °C. The decrease in the thermal transition temperature for rMBP*Pf*Cox11Ct supports the binding of copper to the protein (Fig. 8).

### Chicken IgY antibodies against rMBPPfCox11Ct and the peptide detect the recombinant and native PfCox11 protein

**Fig. 9:**
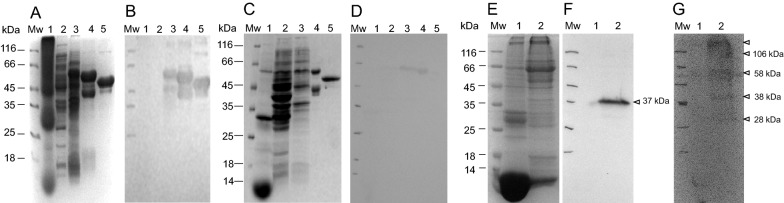
Detection of *Plasmodium* spp. Cox11 and rMBP*Pf*Cox11Ct with chicken anti-rMBP*Pf*Cox11Ct IgY and chicken anti-KIQF{Abu}F{Abu}EEQMLNAKEEM peptide IgY. A 12.5% SDS-PAGE reducing gel stained with Coomassie blue (**A**, **C**) and the same proteins on an identical gel electrophoretically transferred to nitrocellulose (**B**, **D**) and probed with **B** chicken anti-rMBP*Pf*Cox11-Ct IgY or **D** chicken anti-KIQF{Abu}F{Abu}EEQMLNAKEEM peptide IgY. **A**–**D** Mw molecular weight markers; lane 1, uninfected red blood cell lysate; lane 2, untransformed *E. coli* BL21(DE3) host cell lysate; lane 3, *E. coli* expressing rMBP*Pf*Cox11Ct; lane 4, affinity purified rMBP*Pf*Cox11Ct and lane 5, affinity purified recombinant rMPB. **E** Coomassie Blue stained reference gel and proteins transferred to nitrocellulose and probed with **F** chicken anti-*Plasmodium* lactate dehydrogenase peptide IgY and **G** chicken anti-rMBP*Pf*Cox11Ct IgY. **E**–**G** Mw molecular mass marker (Mw); lysates of uninfected (lane 1) and (lane 2) *P. berghei* infected BALB/c mouse red blood cells. Chicken IgY antibodies were detected with rabbit anti-IgY-HRPO secondary antibody and ECL

Antibodies were raised in chickens against the affinity purified rMBP*Pf*Cox11Ct fusion protein and against a peptide in the carboxy-terminus of the protein, coupled to a rabbit albumin carrier. The peptide (KIQF(Abu)F(Abu)EEQMLNAKEEM) was chosen based on hydropathy, antigenicity and surface probability parameters, conservation of the sequence in different species of human infecting *Plasmodium* spp. and the presence of the conserved CFCF motif (see Fig. 1b). The antibodies were isolated and affinity purified [54, 55] using the rMBP*Pf*Cox11Ct protein or peptide coupled to an affinity matrix. The affinity purified rMBP*Pf*Cox11Ct protein was previously shown to be detected by anti-MPB antibodies (Fig. 2b). The affinity purified rMBP*Pf*Cox11Ct protein was separated on an SDS-PAGE gel (Fig. 9a, c) and detected by IgY antibodies against the recombinant protein (Fig. 9b) and antibodies against the synthetic KIQF(Abu)F(Abu)EEQMLNAKEEM peptide (Fig. 9d). The anti-peptide antibodies raised against a synthetic peptide derived from the *Plasmodium falciparum* Cox11 amino acid sequence confirmed the identity of the recombinant rMBP*Pf*Cox11Ct protein (Fig. 9d). All the antibodies detected the 62 kDa recombinant fusion protein and did not detect any *E. coli* host cell proteins or red blood cell proteins (Fig. 9b, d).

Affinity purified chicken IgY antibodies against rMBP*Pf*Cox11Ct were used to probe lysates of uninfected and *P. berghei* infected mouse erythrocytes separated on an SDS-PAGE gel (Fig. 9e) and transferred to nitrocellulose (Fig. 9g). An anti-*Plasmodium* LDH antibody against a conserved *P*LDH common peptide served as a positive control for the assay and detected *Pb*LDH at 37 kDa (Fig. 9f). The antibodies did not detect *E. coli* host cell proteins or mouse red blood cell proteins in the western blot of the gel. The antibodies detected *Plasmodium* proteins at 28 kDa corresponding to the size predicted from the genomic sequence, a 38 kDa protein and a possible dimer and tetramer of 58 and 106 kDa, and a very large protein of undetermined molecular mass (Fig. 9g). This data suggests that the recombinant protein represents the native protein and confirms in silico data indicating that *Plasmodium* spp. parasites express a Cox11 protein [47].

## Discussion

Cox11 is an essential protein in multiple organisms inserting copper into the Cu_B_ site of the Cox1 protein of the terminal mitochondrial respiratory chain enzyme cytochrome *c* oxidase. *Plasmodium* spp. Cox11 has not been studied to date and little is known about both the *Plasmodium* spp. cuprome and assembly of *Plasmodium* cytochrome *c* oxidase. Sixteen proteins of the *Plasmodium* cytochrome *c* oxidase complex have been identified [2]. *Plasmodium* spp. Cox1, which contains the Cu_B_ copper moieties, and Cox3, are encoded by a mitochondrial gene, whilst the remaining proteins, like Cox2 with the Cu_A_ site and Cox11 are encoded by nuclear genes [2, 47]. Here we have begun to characterize *Plasmodium* Cox11 using a recombinant truncated section of the carboxy-terminus of the protein.

The *Plasmodium* spp. Cox11 protein, like the well-characterized Cox11 protein sequences contains a single transmembrane domain (Fig. 1). Tzagoloff et al. [22] proposed that Cox11 is an intrinsic membrane protein in the inner mitochondrial membrane. The C-terminal region of yeast Cox11 can be removed by proteases from mitoplasts indicating its location in the inner mitochondrial membrane space [56] which was confirmed with antibodies [57] and Khalimonchuck et al. [58] proposed an N_in_–C_out_ topology. Given the location of *Plasmodium* spp. cytochrome *c* oxidase and the structural features of the *Plasmodium* Cox11 the *Plasmodium* spp. protein is proposed to have the same location and topology as yeast Cox11.

The transmembrane region of *Pf*Cox11 is followed by a conserved Cys (Cys-60) and a highly conserved 11 amino acid sequence which includes Lys-152 and the CFCF quartet (Cys-155 and Cys-157) seen in Fig. 1A. The cysteines in the CFCF motif map to the same surface accessible position on a loop in the model of *Pf*Cox11-Ct and the Banci et al. [24] *S. meliloti* template model (Fig. 1c). The three cysteines and the Lys residue have been described as essential for Cox11 to insert copper into the Cu_B_ site of cytochrome *c* oxidase [23, 48, 59]. The extensive N-terminal domain, found in yeast, mammalian and plant homologues was shown by proteolytic cleavage studies not to be involved in copper binding [56, 58].

*Plasmodium* spp. Cox11 is predicted to have a size of 26.8 kDa, similar to the 28 kDa originally described for yeast Cox11 [22]. The gene encoding 228 amino acids of the carboxy-terminus of the protein, without the transmembrane region, was cloned and recombinantly expressed as a 19 kDa section of a 62 kDa rMPB fusion protein and affinity purified on an amylose resin (Fig. 2). Banci et al. [24] expressed Cox11 as a hexahistidine His tag fusion protein from five organisms with the best expression being that of the *S. meliloti* protein. Carr et al. [23] found that the *S. cerevisae* Cox11-Ct expressed as a His-Cox11-Ct fusion protein was insoluble, but a thioredoxin fusion protein was soluble. Thompson et al*.* [59] reported poor yields of a His-Cox11-Ct from *Rhodobacter sphaeroides*, but obtained “reasonable yields” of a thioredoxin-Cox11-Ct fusion protein. During the present study there was difficulty expressing the protein as a His tag fusion protein and so a rMPB fusion protein (rMBP*Pf*Cox11Ct) was engineered, expressed and affinity purified.

To compare the *Plasmodium* spp. protein to yeast and bacterial proteins [23, 59], three mutated forms of the protein, two with single C60A and C157A mutations and a double C60A/C157A mutation were expressed and affinity purified. The *Plasmodium* spp. genomes are AT rich and it was consequently not possible in the present study to design suitable primers to modify the Cys-155 residue.

*Plasmodium falciparum* Cox11 was shown to bind copper in a BCA release assay, by atomic absorption spectroscopy, a bacterial host growth inhibition assay, in an ascorbate oxidation inhibition assay and in a thermal shift assay (Figs. 3, 4, 5, 6, 7, 8). The in vitro BCA release assay showed no difference in copper binding between the wild type and the C60A mutant, but there were minor, but not significant, differences between the wild type and the C157A or C60A/C157A double mutant (Fig. 3). When the wild type protein was expressed with additional copper in the medium, more copper was bound to the protein as shown by a 50% increase in absorbance values (Fig. 5). The wild type and the C60A mutant showed little difference in copper binding whilst the C157A and the C60A/C157A mutants bound significantly less copper (Fig. 5). The same trend was observed in the growth pattern of *E. coli* host cells expressing the native and mutated proteins in the presence of growth inhibiting (toxic) concentrations of copper and the ascorbate inhibition assay (Figs. 6, 7). The data indicate that Cys-60 is not important for copper binding in vitro or when the protein is in an in vivo environment, while Cys-157 is essential. The same finding has been reported for *R. sphaeroides* Cox11 [59]. However, though the Cox11 Cys-60 amino acid is not necessary for Cu binding, the amino acid is essential for the insertion of Cu into the Cu_B_ site of Cox1 of cytochrome *c* oxidase in yeast and bacteria [23, 59]. Though we were not able to mutate the Cys-155 amino acid it is very likely that the three cysteines, *P. falciparum* Cox11 Cys-60, 155 and 157 residues, like the same cysteines in Cox11 from yeast and bacteria are essential for physiological function [23, 48].

Mutations of several yeast Cox11 amino acid residues have shown that Lys-205, found three amino acid residues N-terminal to the CFCF motif is essential for a functional cytochrome *c* oxidase [48]. All *Plasmodium* spp. genomes sequenced to date and a large number of other sequences from multiple species (personal observation) have conserved amino acid residues either side of the CFCF motif including the lysine residue (Fig. 1). Met-224, indicated as essential for the yeast Cox11 sequence, is not present in any *Plasmodium* homologues (Fig. 1b), *Babesia bovis* or *Theileria parva* Cox11 proteins (Fig. 1a) [23]. Banting and Glerum [48] found that mutations of other residues of bacterial Cox11, for example, Tyr-192 and Val-226 impaired Cox11 function. The Tyr-192 is present in all *Plasmodium* homologues (*P.* Tyr-139) and the Val-226 in *S. cerevisae* (Fig. 1a) is present in *Plasmodium vivax*, *Plasmodium knowlesi* and all rodent *Plasmodium* Cox11 sequences (9 amino acids Ct to box d in Fig. 1b). Interestingly *P. adleri, P. bilicollins, P. blacklocki, P. gaboni, P. reichenowi, P. praefalciparum*, *P. gallinaceum* and *P. falciparum* (in 16 different isolates) all have an isoleucine (*Pf*Cox11 Ile-173) in place of valine in homologues of Cox11 from other species [47]. There is a single base difference at the 5' end of three codons coding for either valine (G) or isoleucine (A), indicating how the change in a single base gives rise to the alternate amino acid, independent of which of the three different codons are employed. These findings strongly support Banting and Glerum’s [48] observation and confirm that in the position occupied by Val-226, a hydrophobic amino acid is essential and can be either valine or isoleucine. Banting and Glerum [48] found that replacing Val-226 with a tryptophan produced a dysfunctional Cox11 showing that a more bulky hydrophobic side chain influences the physiological activity of Cox11.

The expression of *P. falciparum* Cox11 in vivo is indicated by the functionality of *Plasmodium* cytochrome *c* oxidase and mRNA expression data [47, 49, 50]. Affinity purified antibodies raised against the recombinant protein and against a peptide sequence derived from the amino acid sequence of the protein detected the recombinant protein (Fig. 9b, 9d). These antibodies detected the native protein in a lysate of blood stage parasites (Fig. 9g). This confirms that the recombinant protein shares structural features with the native protein and provides physical evidence for the expression of the native protein by the parasite. The antibodies detected five proteins in the parasite lysate. The native protein is 26.8 kDa and a protein of 28 kDa was detected in the western blot (Fig. 9g). The larger protein at 58 kDa may represent a possible Cox11 homodimer or a heterodimer of Cox11 and Cox19 (26.8 and 26.1 kDa) as the two have been shown to interact and co-purify [60]. The 108 kDa protein may represent a Cox11 homotetramer, while the largest protein could represent Cox11 interacting with proteins of the cytochrome *c* oxidase complex. The western blot could be probed with antibodies against the relevant proteins to support or disprove this suggestion.

Yeast Cox11 can obtain copper from Cox17 [15]. Given the structural similarities between the *Plasmodium* and yeast proteins shown in this study and by Choveaux et al. [16], it is suggested that *Plasmodium* Cox11 would also obtain copper from Cox17, which will be evaluated. Yeast Cox11 inserts copper to the Cu_B_ site on the Cox1 protein of the cytochrome *c* oxidase [24, 48, 61]. Expression data for mRNA indicates that *Pf*Cox17, *Pf*Cox11 and *Pf*Cox1 are expressed during the sporozoite, trophozoite and gametocyte stages of parasite development [49] and interestingly expression levels of *Pf*Cox17 and *Pf*Cox11 peak at 30 h and *Pf*Cox1 at 40 h post erythrocytic invasion [50]. This suggests an important role for complex IV during the development from trophozoites to schizonts in red blood cells to be investigated further.

This study has confirmed: the presence of native Cox11 in *Plasmodium* spp., that *Pf*Cox11 binds copper in vitro or an in vivo* E. coli* environment and that Cys-157 is essential for copper binding in vitro*.* The gene for *PfCox11* has a mutagenesis index score and a mutant fitness score of 0.145 and −2.887 which are similar to those of two *Plasmodium* spp. glycolytic enzymes lactate dehydrogenase and glyceraldehyde-3-phosphate dehydrogenase suggesting the essentiality of the protein for optimal parasite growth [62]. *Plasmodium* spp. obtain most of their metabolic energy from glycolysis which would explain the essentiality of the glycolytic enzymes [63, 64]. There is uncertainty about the role of the *Plasmodium* spp. electron transport chain and cytochrome *c* oxidase or complex IV in the metabolism of *Plasmodium* spp. [4]. What is known is that inhibitors of complex IV depolarize the membrane potential (Δψ) across the inner mitochondrial membrane and reduce oxygen consumption [65, 66]. Further studies of *Plasmodium* Cox11 will contribute to the understanding of *Plasmodium* copper metabolism and the assembly and metabolic role of *Plasmodium* spp. cytochrome *c* oxidase and the electron transport chain.

## Data Availability

The datasets used and/or analysed during the current study are available from the corresponding author on reasonable request.

## Abbreviations

Abu: Alpha amino butyric acid
ATOX-1: Antioxidant Protein 1
BCA: Bicinchoninic acid
CCS: Copper Chaperone for Superoxide dismutase
Cox: Cytochrome *c* oxidase
Ctr1: Copper transporter 1
ECL: Enhanced chemiluminescence
HRPO: Horse radish peroxidase
rMBP: Recombinant maltose binding protein
rMBP*Pf*Cox11Ct: Recombinant maltose binding protein *Plasmodium falciparum* Cox11 carboxy-terminus
